# Bidirectional Selector Utilizing Hybrid Diodes for PCRAM Applications

**DOI:** 10.1038/s41598-019-56768-2

**Published:** 2019-12-27

**Authors:** Yi Shuang, Shogo Hatayama, Junseop An, Jinpyo Hong, Daisuke Ando, Yunheub Song, Yuji Sutou

**Affiliations:** 1 Department of Materials Science, Graduate School of Engineering, Tohoku University, 6-6-11 Aoba-yama, Sendai, 980-8579 Japan; 2 Department of Electronic Engineering, Hanyang University, 17 Haengdang-dong, Seongdong-gu, Seoul 133-791 Korea; 3 Department of Physics, Hanyang University, Seoul, 04763 Korea

**Keywords:** Electronic devices, Information storage, Surfaces, interfaces and thin films

## Abstract

Three-dimensional crossbar technology has been of great significance for realizing high density and multiple terabytes of data storage in memory devices. However, to further scale down the size of memory devices, a selector exhibiting nonlinear electrical properties should be in series with a memory layer in case of unwanted sneak current disturbance. Conventional selectors usually utilize a complicated multilayer structure to realize the high nonlinearity of current, which might be incompatible with certain manufacturing processes or limit the scalability of memory. Herein, we propose a simple heterojunction diode using an n-type oxide semiconductor, specifically, InGaZnO_4_ (IGZO), and a p-type phase change material (PCM), specifically, N-doped Cr_2_Ge_2_Te_6_ (NCrGT), to realize self-selective performance. The electrode/IGZO/NCrGT/plug-electrode structure with an IGZO/NCrGT pn diode and NCrGT/plug-electrode Schottky diode can realize bidirectional, self-selective phase change random access memory (PCRAM) for either amorphous or crystalline NCrGT. The approximate equilibrium energy band diagrams for the IGZO/NCrGT pn junction and the IGZO/NCrGT/W hybrid junction were proposed to explain the possible conduction mechanism. We demonstrated that hybrid diode-type PCM memory exhibits both selectivity and resistive switching characteristics. The present findings offer new insight into selector technology for PCM.

## Introduction

Nonvolatile memory (NVM) plays an essential role in our lives due to the popularization of portable electronic devices such as cellphones and laptops. However, conventional two-dimensional (2D) flash memory based on storing charges is now approaching its physical limitations according to Moore’s law^1^. In recent years, 3D flash memory has been developed and manufactured to replace the 2D type^2^. As another candidate for future NVM, three-dimensional (3D) crossbar technology was introduced and manufactured by Intel Corporation and Micron Technology, Inc^3^. In this array, phase change random access memory (PCRAM) is used as a memory component because it can meet overall requirements such as rapid switching speed, good data retention, high endurance, high scalability, and low cost^4–6^. For decades, many studies have attempted to improve the performance of PCRAM, namely, by reducing operation energy and enhancing endurance^7,8^. To achieve higher storage density, 3D crossbar technology was exploited to deliver multiple terabytes of storage on a single chip^9^. In an ideal 3D crossbar structure, the memory operation is conducted on selected cells, with other cells unselected. The resistance of phase change materials (PCMs) in the crystalline phase is generally small, and the sneak current will be introduced in 3D crossbar memory arrays, which greatly degrades the read accuracy and raises the total power consumption^10^. Thus, a nonlinear circuit element, such as a selector, should be serially connected with a memory element to suppress parasitic leakage current at small voltage bias.

Several categories of selector elements have been widely investigated to achieve high-level integration of PCRAM, including Si-based or oxide-based diodes^11^, the ovonic threshold switch (OTS)^12^, and mixed electronic and ionic conduction (MIEC)^13^. The epitaxial growth of a single crystalline Si pn diode was first demonstrated as a selector for PCRAM, exhibiting excellent nonlinear current-voltage (*I*-*V)* characteristics and a high on/off current ratio of around 10^8^ ^11^. OTSs are generally composed of amorphous chalcogenide materials and show volatile, nonlinear *I*-*V* switching behavior. An OTS has been successfully integrated into a one-selector-one-resistor (1S1R) PCRAM array^12^. Cu-containing MIEC materials were confirmed to be suitable for use in large 3D PCRAM arrays due to their potential for combining multi-level cells with 3D stacking^14^. However, several significant issues exist regarding the scalability and practical application of PCRAM. Due to their low crystallization temperatures, OTS materials usually cannot endure a high-temperature environment^15^; MIEC-based selectors are likely to degrade at high current stress (i.e., undergo Cu accumulation failure)^16^. Si pn diode growth requires a high fabrication temperature at 700 °C for dopant activation that is not compatible with the sub-400 °C back-end-of-line (BEOL) process for fabricating integrated circuits^17,18^, and its complex multilayer structure is not suitable for 3D integration^19^. As a replacement for the traditional 1S1R or one-diode-one-resistor (1D1R) device configuration, simple self-selected memory cells based on self-rectifying *I*-*V* characteristics have been proposed in metal-oxide resistive RAM, for example, the Ti/TiO_2_/Pt metal-insulator-metal sandwich structure^20^ and the p^+^-Si/n-ZnO heterostructure-based 1D1R memory^21^. Nevertheless, few efforts have aimed to utilize PCMs for the design of self-selected memory structures since their crystalline phase is generally metallic in nature^22^, which indicates that PCMs cannot form a rectified *I*-*V* behavior with a semiconductor layer or metal electrode in their crystalline phase. Recently, our group has proposed a Cr_2_Ge_2_Te_6_ (CrGT)-type PCM that shows a unique phase transition from a low-resistance amorphous phase to a high-resistance crystalline phase^23–25^. By doping a certain amount of nitrogen into CrGT, there is almost no difference between the resistance exhibited in the amorphous and crystalline phases, while a 3-order resistance contrast can be obtained in a memory cell using N-doped CrGT (NCrGT) and a W electrode. Such a 3-order resistance contrast can be realized by the contrast of contact resistance between the high-contact resistance of amorphous NCrGT/W and the low-contact resistance of crystalline NCrGT/W^26^.

Herein, we propose a pn diode structure composed of n-type InGaZnO_4_ (IGZO) and p-type N-doped Cr_2_Ge_2_Te_6_ (NCrGT) PCM, where NCrGT exhibits semiconductor-like behavior in both phases and reversible switching performance^26^. We confirmed that rectification pn junction behavior is achieved in both the amorphous and crystalline phases of NCrGT. Furthermore, we demonstrated a hybrid diode configuration composed of IGZO/NCrGT pn junction and NCrGT/W Schottky junction, enabling a bidirectional selector. The switching performance of this bidirectional self-selected PCRAM was successfully operated. The realization of the NCrGT-based self-selected PCRAM offers a new opportunity for designing a highly scalable 3D cross-point PCRAM array.

## Results and Discussion

### Band gap and electrical properties of IGZO and NCrGT films

**Figure 1:**
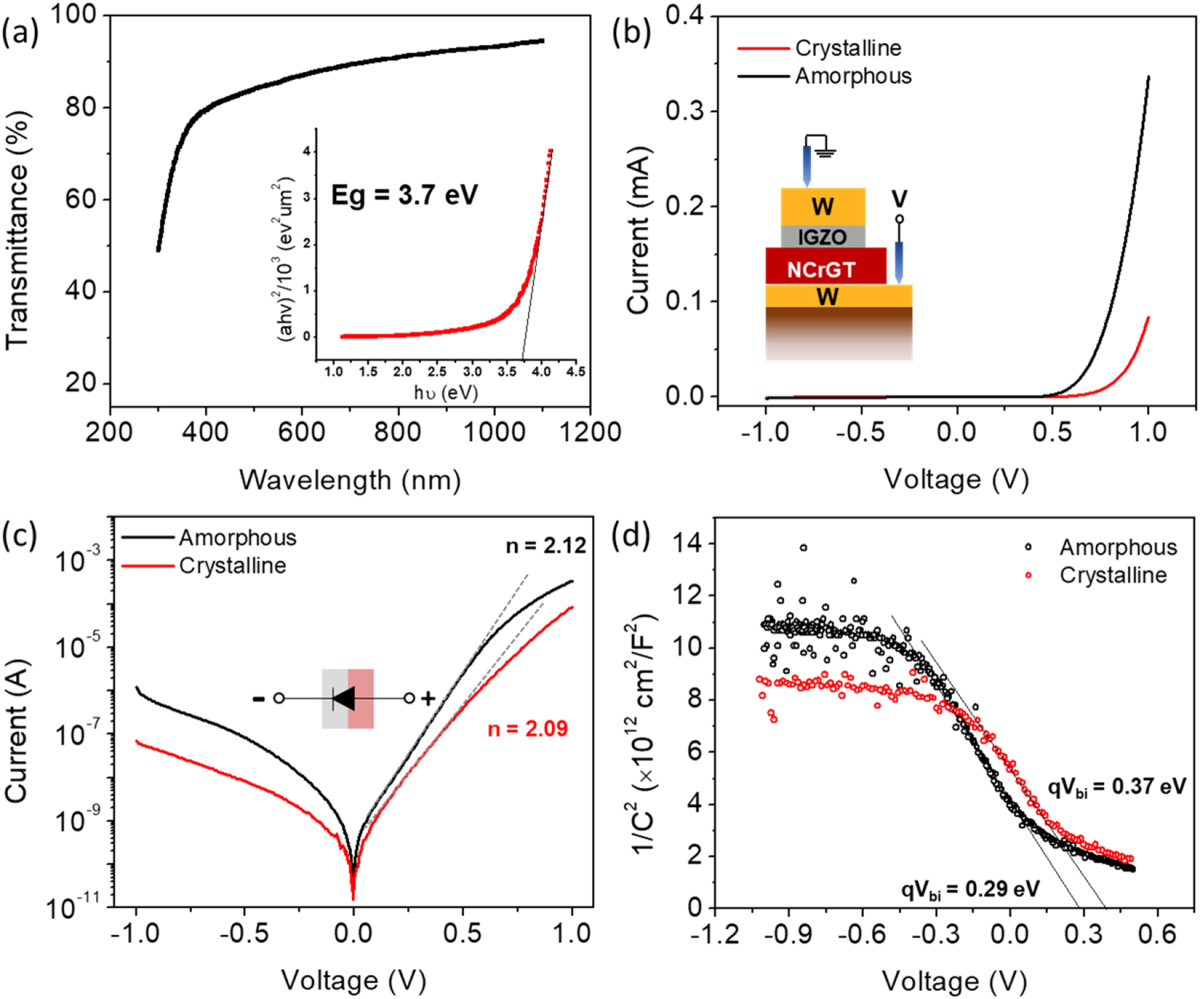
(**a**) The transmittance of IGZO as a function of wavelength. Inset is the absorption spectra of IGZO. (**b**) *I*-*V* curves of IGZO/NCrGT heterojunction; inset shows a schematic diagram of the diode structure. (**c**) Semi-logarithmic *I*-*V* curves of (**b**); inset shows the current flow direction (the gray and pink blocks represent IGZO and NCrGT, respectively). (**d**) 1/*C*^2^-*V* curves to evaluate the built-in voltage of the IGZO/NCrGT junction.

Figure 1(a) shows the optical transmittance of the present IGZO in the visible light range. The absorption coefficient (α) can be calculated from the following Eq. (1) ^27^:

1 $${\rm{\alpha }}=1/d\,\mathrm{ln}(1/T),$$

where *d* is the thickness of the IGZO film, and *T* is the transmittance. The optical energy bandgap (*E*_g_) of the IGZO film was determined from the value of α according to Tauc’s model expressed by the following equation^27^:

2 $${\rm{\alpha }}h\nu ={\rm{A}}{(h\nu -{E}_{g})}^{{\rm{n}}},$$

where *h* is Plank’s constant, *ν* is the frequency, A is a constant, and n = 0.5, indicating the direct semiconductor nature of IGZO. The plot of (α*hv*)^2^ against *hν* is shown in the inset of Fig. 1(a), and the *E*_g_ of our IGZO was estimated to be 3.7 eV, which is comparable to the value in the literature^28^. X-ray diffraction (XRD) measurements revealed that the as-deposited and annealed (330 °C and 380 °C) NCrGT films were in an amorphous phase and a Cr_2_Ge_2_Te_6_ crystalline phase with a rhombohedral structure with R$\overline{3}$ symmetry, respectively, and the as-deposited IGZO film was in an amorphous phase (see Supplementary Fig. S1). The *E*_g_s of the NCrGT in the amorphous and crystalline phases were measured by spectrophotometer. The absorption coefficients of the amorphous and crystalline phases in the NCrGT were evaluated from transmittance measurements of films with different film thickness (see details of the method in Supplementary Fig. S2)^29^. The *E*_g_s of the NCrGT in the amorphous and crystalline phases were determined to be 0.5 and 0.8 eV, respectively. The electrical properties of both IGZO and NCrGT films were determined by Hall-effect measurements. The present IGZO was confirmed to be an n-type semiconductor that possessed resistivity (*ρ*) of 1.5 × 10^4^ Ω·cm, the carrier density (*n*) of 1.1 × 10^14^ cm^−3^, and the mobility (*μ*) of 3.62 cm^2^/V·s (Supplementary Table S1). Moreover, we have already reported that NCrGT is a p-type semiconductor in both amorphous and crystalline phases and shows the following electrical properties: *ρ* = 6.4 Ω·cm, *n* = 1.6 × 10^20^ cm^−3^, and *μ* = 0.020 cm^2^/V·s in the amorphous phase, and *ρ* = 7.6 Ω·cm, *n* = 0.7 × 10^20^ cm^−3^, and *μ* = 0.036 cm^2^/V·s in the crystalline phase (Supplementary Table S1)^26^.

### *I*-*V* characteristics of W/IGZO/NCrGT/W layered structure

Figure 1(b) shows the *I*-*V* characteristics of a W/IGZO/NCrGT/W heterojunction diode analyzed by a semiconductor parameter analyzer, and the device structure is shown in the inset. The forward bias was applied to the W bottom electrode. To rule out the influence of a Schottky diode between W/IGZO or NCrGT/W interfaces, we also examined the *I*-*V* measurement for the W/IGZO/W and W/NCrGT/W structures, where the contact sizes for the top and bottom electrodes were the same in both structures (see Supplementary Fig. S3). Both structures exhibited a slightly nonlinear *I*-*V* behavior with the current flow much higher than the on-current of the pn diode, indicating that the *I*-*V* characteristics shown in Fig. 1(b) were dominated only by the interface conduction of the IGZO/NCrGT pn heterojunction. Figure 1(c) shows the semi-logarithmic *I*-*V* characteristics. It is obvious that both the amorphous and crystalline NCrGT can exhibit a rectification property upon contact with n-type IGZO. The nonlinearity calculated from the current ratio between forward and reverse biases at voltages of 1 V and −1 V was estimated to be more than 10^3^. The ideality factor was determined to be 2.12 for the amorphous phase and 2.09 for the crystalline phase from the slope of the linear part of the forward log *I*-*V* curve, indicating an ideal pn diode with a carrier recombination–dominant process^30^. The built-in voltage (*V*_bi_) of the heterojunction can be evaluated via capacitance-voltage (*C*-*V*) measurement by neglecting the influence of the interface states of the diode on the capacitance. The working frequency was set to be 1 MHz in the *C*-*V* measurement. Figure 1(d) shows the 1/*C*^2^ vs. *V* plots in the W/IGZO/NCrGT/W heterojunction diode with amorphous or crystalline NCrGT, indicating that *V*_bi_ was equal to 0.29 and 0.37 V, according to the abscissa intercepts for the amorphous and crystalline NCrGT, respectively. The results indicate the realization of a pn junction between n-type IGZO and p-type NCrGT stack without doubt. The *I*-*V* behaviors of the W/IGZO/NCrGT/W may be of great benefit for selector operation in both the amorphous and crystalline NCrGT states.

**Figure 2:**
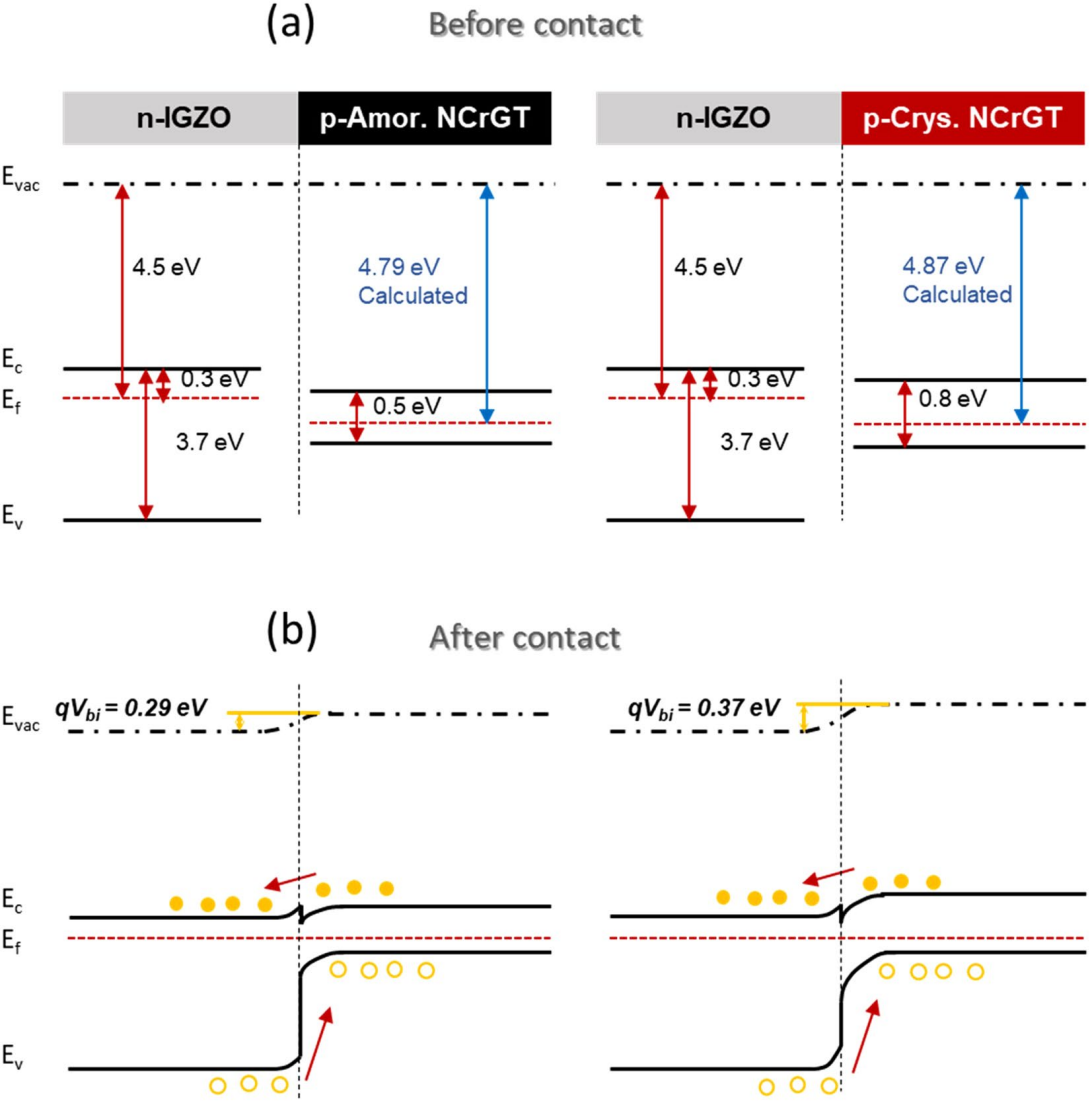
Band diagram of IGZO/NCrGT heterojunction. (**a**) Before contact. (**b**) Equilibrium state after contact. Left and right images indicate the band diagrams for amorphous and crystalline NCrGT, respectively.

Equilibrium band diagrams of the IGZO/NCrGT heterojunction diode are shown in Fig. 2. According to the literature, the conduction band minimum (*E*_c_) and Fermi level (*E*_f_) of IGZO are 4.2 eV and 4.5 eV, respectively, with respect to the vacuum level^31^. Both amorphous and crystalline NCrGT films showed a p-type semiconductor characteristic; therefore, *E*_f_ would be positioned near the valence band maximum (*E*_v_) (Fig. 2(a)). After the formation of a diode contact, as shown in Fig. 2(b), the IGZO exhibited a strong mismatch with the NCrGT because of its much larger bandgap, resulting in a conduction band spike. The built-in potential *qV*_bi_ is expressed by the Fermi level of the p-type (*E*_fp_) and n-type (*E*_fn_) semiconductors as follows:

3 $$q{V}_{{\rm{bi}}}={E}_{{\rm{fn}}}-{E}_{{\rm{fp}}},$$

where *q* is the elementary charge. From the measured *V*_bi_ value, as shown in Fig. 1(d), the *E*_f_ of the amorphous and crystalline NCrGT could be estimated to be approximately 4.79 and 4.87 eV, respectively. The estimated *E*_f_ of the crystalline NCrGT (4.87 eV) was comparable to the *E*_f_ of 4.92 eV observed by ultraviolet photoelectron spectroscopy (see Supplementary Fig. S4). However, the estimated *E*_f_ of the amorphous NCrGT (4.79 eV) was slightly smaller than the observed *E*_f_ of 4.9 eV, which may occur if more defect states are present between the amorphous NCrGT and IGZO^32^. The lower *qV*_bi_ also led to the increment of reverse current in the amorphous NCrGT/IGZO diode, as shown in Fig. 1(c) ^33^.

### *I-V* characteristics of W/IGZO/NCrGT/W-plug-type device

**Figure 3:**
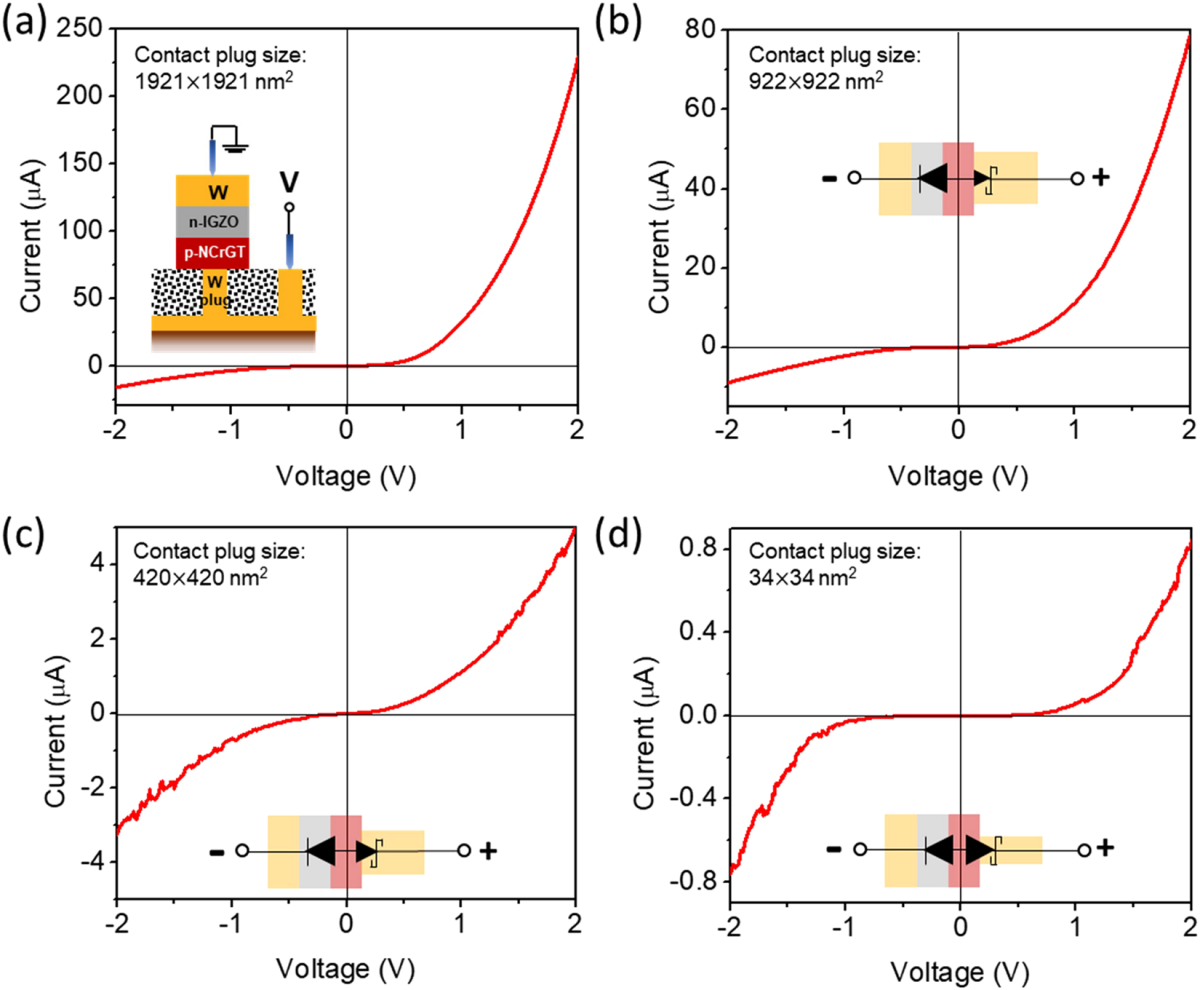
*I*-*V* curves of plug-type devices measured from various plug contact areas with a square shape, *d × d* nm^2^, in a crystalline NCrGT state. (**a**) *d* = 1921 nm, and the inset is the device structure diagram, (**b**) *d* = 922 nm, (**c**) *d* = 420 nm, (**d**) *d* = 34 nm. The inset in (**b**–**d**) is a schematic diagram of a hybrid-type diode with an indication of the current flow direction, where the gray, red, and gold blocks represent IGZO, NCrGT, and W, respectively.

Figure 3 displays the *I*-*V* characteristics of plug-type devices with variable plug contact areas, *d* × *d* nm^2^, in the crystalline NCrGT state. The schematic diagram of the plug-type device (W/IGZO/NCrGT/W-plug) is shown in the inset of Fig. 3(a), and all NCrGT layers here were annealed up to 330 °C to obtain a crystalline phase. The forward voltage bias was applied to the W-plug bottom electrode. We found that the nanoscale contact between the NCrGT and the W electrode resulted in a Schottky-like junction between them. When *d* = 1921 nm, the *I*-*V* curve exhibited an asymmetric property, showing negligible Schottky contact behavior due to the large contact area, namely ohmic-like contact. With the decrease of *d*, the forward current declined, and the *I*-*V* curve exhibited a symmetric-like property, indicating that not only the pn diode of IGZO/NCrGT but also the Schottky diode dominated the *I*-*V* characteristics of the plug-type device, as shown in the insets of Fig. 3(b–d). When the value of *d* decreased to 34 nm, an almost perfectly symmetric ambipolar *I*-*V* curve was achieved, with the current scaling down to a nano-ampere order. The forward current kept decreasing with the decrease of the contact area of the NCrGT/W-plug. Meanwhile, in the case of large *d*, the leakage current level of the plug-type device in reverse bias was about one order of magnitude higher than that of the pn diode indicated in Fig. 1(c), which implies that there was a remarkable tunneling current flowing at the pn junction in the plug-type device.

### Contribution of the schottky diode in a W/IGZO/NCrGT/W-plug-type device

**Figure 4:**
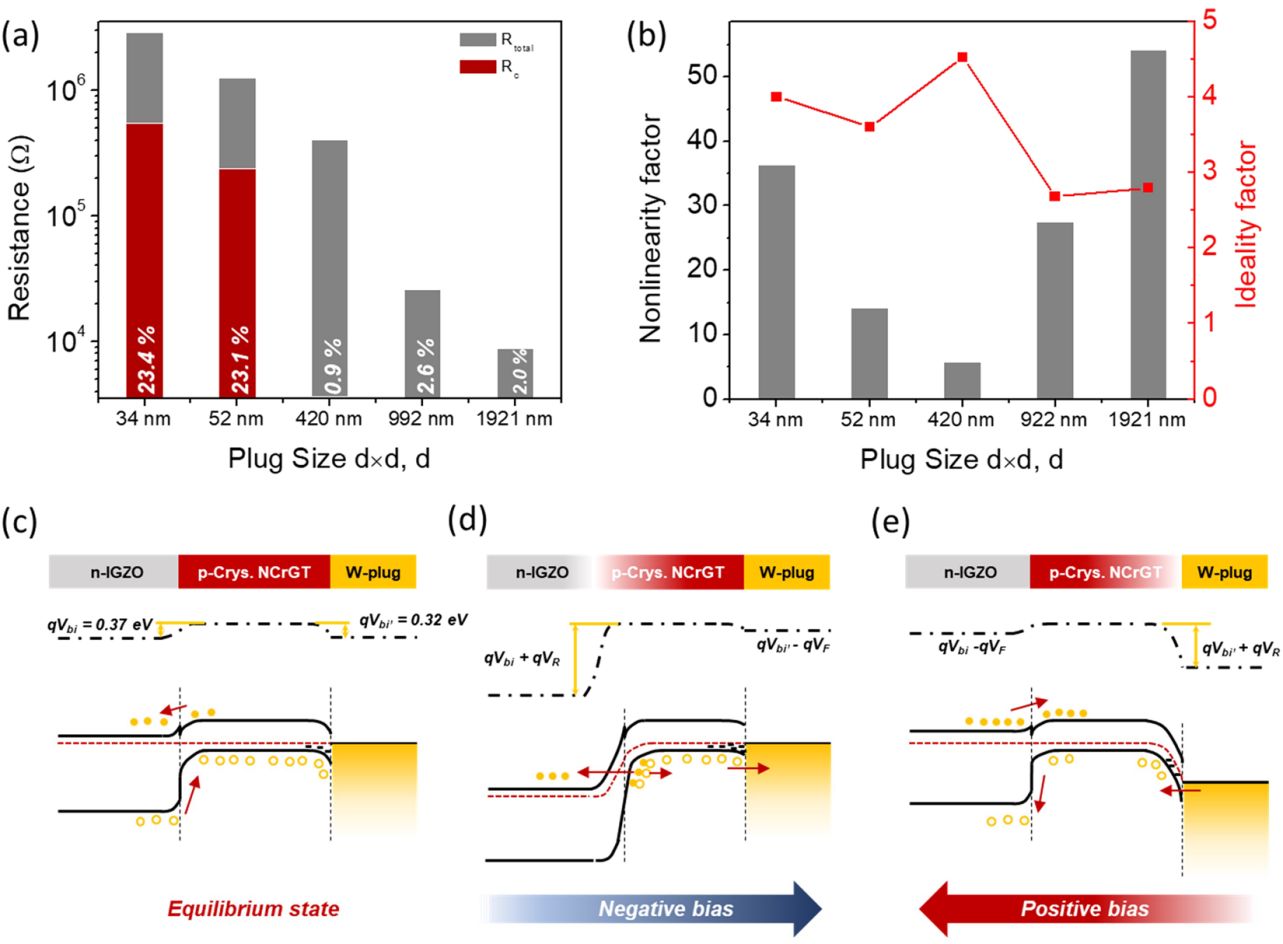
(**a**) Dependence of W-plug size, *d*, on the ratio of the Schottky contact resistance to the total resistance of the device. (**b**) Nonlinearity factor and ideality factor under forward bias current as a function of *d*. Band alignment of IGZO/NCrGT/W-plug hybrid structure at (**c**) equilibrium state, (**d**) negative voltage bias, and (**e**) positive voltage bias.

To understand the behavior of the Schottky diode in the NCrGT/W-plug contact, we calculated the ratio of the Schottky contact resistance to the total resistance. The total resistance was determined at a read voltage of 2 V. The contact resistivity (*ρ*_c_) was calculated by using the circular transfer length method (CTLM) (see Supplementary Fig. S5). The contact resistance (*R*_c_) between the NCrGT and the W-plug could be estimated according to the equation *R*_c_ = *ρ*_c_/A, where A is the contact area, which is equal to *d* × *d*. Figure 4(a) shows the calculated total resistance (*R*_total_, gray column) and the Schottky contact resistance of the NCrGT/W-plug (*R*_c_, red column). *R*_c_ is clearly shown to occupy merely a small fraction of *R*_total_ at larger contact sizes (*d* ≥ 420 nm), whereas at *d* = 34 nm and 52 nm, *R*_c_ accounted for 23% of *R*_total_. This indicates that the influence of the contact resistance on the total device resistance became larger as the contact area between the NCrGT and the W-plug bottom electrode decreased. To describe the conduction mechanism in this hybrid structure, the ideality factor was obtained from the slopes of the semi-logarithmic plots in the forward direction. At *d* = 1921 nm and 922 nm, the ideality factor was calculated to be 2.8 and 2.7, respectively, exhibiting only a slight deviation from the ideal carrier recombination–dominant pn diode behavior, as indicated in Fig. 1(c). However, the ideality factor became higher with decreasing *d* since the influence of the Schottky contact between the NCrGT and the W-plug increased (Fig. 4(b)). The nonlinearity under the forward bias current evaluated from the ratio of current at 1 V and 2 V in devices with various *d* is also summarized in Fig. 4(b). In a range from 1921 to 420 nm, nonlinearity was degraded with decreasing *d*. The largest ideality factor and lowest nonlinearity were obtained at *d* = 420 nm, which might be due to competition between the pn junction and the Schottky diode current at this critical size dimension. When the value of *d* further decreased, the nonlinearity improved again. As a result, a nonlinearity of 36 was achieved in the bidirectional nonlinear *I*-*V* curve at *d* = 34 nm. Here, we discuss the conduction mechanism of the two-diode hybrid structure observed in the plug-type device with a small *d* value. Figure 4(c) shows the equilibrium band diagram without any voltage bias applied. There was a built-in voltage in both the n-IGZO/p-NCrGT and p-NCrGT/W-plug Schottky junctions. When a negative voltage bias was applied to the device, the pn diode was connected in a reverse-bias condition, and the energy barrier was too high for electrons to transfer from the n-IGZO conduction band to the p-NCrGT conduction band. Therefore, the impact ionization occurred at the reverse-biased junction, generating new electron-hole pairs, which could act as new carriers^34^. At the same time, the Schottky diode of the p-NCrGT/W-plug was connected in a forward-bias condition, and, therefore, the holes could easily diffuse across the small built-in potential, as shown in Fig. 4(d). On the contrary, when positive voltage bias was applied, the built-in potential of the pn junction was weakened, resulting in a diffusion of carriers at the junction. The hole carriers could only tunnel from the W-plug to the p-NCrGT at the Schottky junction through the higher barrier in a reverse-bias condition, as shown in Fig. 4(e). Consequently, the rectifying behavior in both forward and reverse biases (i.e., symmetric rectifying behavior) was suggested to be obtained in this IGZO/NCrGT hybrid diode structure.

### Resistive switching memory characteristics in a W/IGZO/NCrGT/W-plug-type device

**Figure 5:**
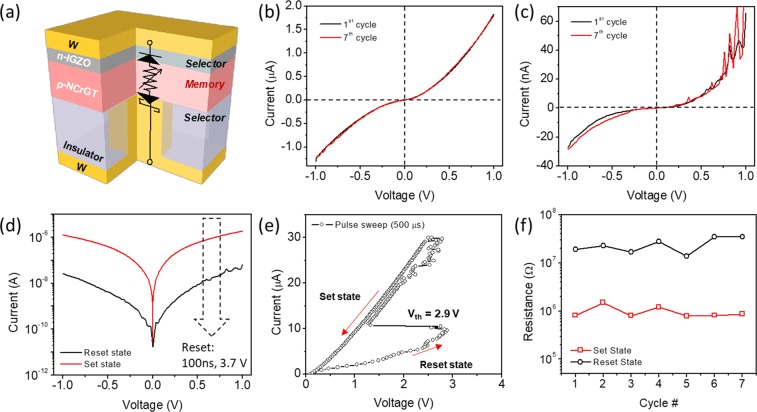
(**a**) Schematic diagram of the proposed bidirectional self-selective PCRAM device. (**b**) *I*-*V* characteristics of a low-resistance set (crystalline NCrGT) state obtained at the first (black) and seventh (red) cycle measurements. (**c**) *I*-*V* characteristics of a high-resistance reset (amorphous NCrGT) state obtained at the first and seventh cycle measurements. (**d**) Semi-logarithmic *I*-*V* curve obtained in the set (red) and reset (black) states obtained at the first and seventh cycle measurements. (**e**) Typical threshold switching behavior of the device; the initial state was the reset state. (**f**) Cyclic resistive switching memory performance of the device.

The resistive switching memory operation was demonstrated using a plug-type device with *d* = 34 nm, which shows a symmetric rectifying behavior and is favorable for low thermal energy operation. As shown in Fig. 5(a), a pulse generator was connected to the device in series to apply a nanosecond-order short voltage pulse, and a semiconductor analyzer was used to measure the *I*-*V* characteristics of two different states, that is, the set and reset states. Before the resistive switching memory operation, a current sweep from 0 to 30 μA was applied to the device to obtain a sufficiently low resistance state. The *I*-*V* characteristic of the set state showed nonlinear and symmetric behavior, as shown in Fig. 5(b). Then, the device was reset by applying a 100 ns voltage pulse of 3.7 V, which increased the device resistance, which was dominated by the Schottky contact resistance between W and NCrGT^26^. The *I*-*V* curve of the reset state also showed nonlinearity in both forward and reverse bias, as shown in Fig. 5(c). The semi-logarithmic *I*-*V* curves of the set and reset states are shown in Fig. 5(d). It is obvious that the current declined by around two orders of magnitude after the reset operation. These results indicate that the W/IGZO/NCrGT/W-plug-type device can show both bidirectional self-selective and resistive switching memory functionalities. However, the *I*-*V* curve of this device showed large ideality factors of 4.04 and 3.30 in the set and reset states, respectively, where the values were obtained by fitting the linear part of the semi-logarithmic *I*-*V* curves. These values are larger than those of the initial state of the device (Fig. 3(d)), indicating the degradation of the diode characteristic. According to the Ellingham diagram, ZnO is less stable than CrO_x_ and GeO_x_; therefore, the oxygen in IGZO can react with the Cr and Ge in NCrGT, especially during the joule heating process of the set and reset operations, resulting in the formation of an interfacial reaction layer between IGZO and NCrGT. The interlayer may introduce more defects, suppressing an ideal carrier diffusion process at a pn diode junction^35,36^. In addition, the mobility and carrier density of IGZO might increase by thermally annealing it at more than 250 °C, due to the reduction of trap states by its random amorphous structure^37^. Therefore, there is also a risk that a similar thermal annealing effect will occur in our IGZO layer when applying a higher current or voltage pulse. The reasons above may explain the degradation of the diode behavior. The reversibility of the resistive switching operation was also demonstrated, as shown in Fig. 5(f). The device resistance in both the set and reset states was read at 1 V. In this study, for the set operation, a current pulse sweep from 0 to 30 μA was applied to the device, that is, the set state was obtained via typical ovonic threshold switching, as indicated in Fig. 5(e). As the sweeping current was increased, the voltage gradually increased in the initial stage and then snapped back at a threshold voltage (*V*_th_) of 2.9 V. After the resistive switching, the resistance dropped to a low resistance set state, indicating the crystallization of the NCrGT. For the reset operation, a 100 ns voltage pulse of 3.7 V was applied to the device. Even after seven cycles of switching, the *I*-*V* characteristics of the set and reset states were confirmed not to have deteriorated and to be consistent with that in the first cycle, as shown in Fig. 5(b,c). However, after more switching cycles, the device failed to be switched to the set state, which may be due to an excessive interfacial reaction between the IGZO and NCrGT layers induced by large unwanted joule heating energy upon the switching operation. To reduce the set/reset current, we fabricated devices with the same structure by using TiN instead of W as a contact in order to enhance the thermal efficiency of the memory cell, as TiN has much lower thermal conductivity^38,39^. The TiN-plug-based device exhibited a lower current pulse (20 μA) for the set process, indicating that Joule heating energy can be effectively declined by using a TiN-plug device (see Supplementary Fig. S6). However, the endurance performance still cannot be further improved, even by lowering the Joule heating effect (see Supplementary Fig. S6). Therefore, the interface diffusion of Cr and Ge might be activated much more easily by the Joule heating effect than we expected. We summarized that the weakness of the low stability of IGZO is fatal for achieving much better cyclability. Therefore, further nonlinearity or selectivity and cyclability can be improved by carefully selecting the n-type oxide materials, for instance, oxides with higher stability than CrO_x_ and GeO_x_, such as TiO_x_ or Nb-doped TiO_x_ which are also famous transparent conductive oxides for various electrical or optical applications^40,41^.

## Conclusions

We conducted a feasibility study of a self-selective pn diode-type PCRAM device using n-IGZO and NCrGT PCM with p-type conductivity in both the amorphous and crystalline phases. The device exhibited diode-like rectifying behavior for both amorphous and crystalline phases of NCrGT. Furthermore, a bidirectional self-selective property was found to be obtained in a device with a plug-type W electrode. The device composed of a W/IGZO/NCrGT/W-plug structure showed not only the n-IGZO/p-NCrGT pn diode property but also the NCrGT/W-plug Schottky diode property by reducing the size of the contact plug. The conduction mechanism of the hybrid diode was reasonably understood from the band alignment of IGZO and NCrGT. We also confirmed that the hybrid diode-type device shows a resistive switching property with a resistance contrast of around two orders of magnitude; simultaneously, the device shows a symmetric rectifying behavior in both the set and reset states, indicating that the bidirectional self-selective PCRAM is possible using n-type oxide and NCrGT PCM. Although the selectivity characteristics of pn- and Schottky diode selectors and the stability at the interface between oxide and NCrGT are still insufficient for practical applications, the strategy of designing the bidirectional and self-selective PCRAM utilizing hybrid diodes may offer us great insight into the development of selector technology and highly scalable 3D crossbar array PCRAM devices.

## Methods

### Thin film deposition and characterization

Both IGZO and NCrGT thin films were deposited onto a precleaned corning glass substrate by using radio frequency (RF) magnetron sputtering at room temperature. The argon gas flow rate was 15 sccm. NCrGT films approximately 40–200 nm thick were deposited by co-sputtering from Ge, Cr, and Te targets under reactive N_2_ gas at the gas flow rate (${f}_{{{\rm{N}}}_{2}}$) of 0.4 sccm. It was confirmed by X-ray photoelectron spectroscopy that the N content of the NCrGT film deposited under ${f}_{{{\rm{N}}}_{2}}$ = 0.4 sccm in our sputtering chamber was 6.5 at.%^26^. The as-deposited NCrGT was then annealed up to 380 °C at a heating rate of 10 °C/min, followed by cooling to room temperature in an Ar atmosphere to obtain a crystalline phase. An IGZO film 40 nm thick was deposited by sputtering from an InGaZnO_4_ compound target under reactive O_2_ gas at a gas flow rate (${f}_{{{\rm{O}}}_{2}}$) of 0.2 sccm. The thickness of the film was measured by atomic force microscopy. The crystal structures of the as-deposited and annealed films were investigated by XRD (Rigaku, ULTIMA). The electrical properties of the films, including resistivity, carrier type, carrier density, and mobility, were evaluated by Hall-effect measurements (Toyo Corp., ResiTest 8400). The transmittance of the films was measured by spectrophotometer (V-630 UV-visible/NIR spectrophotometer, Jasco) to investigate the optical bandgap (*E*_g_) of the NCrGT and IGZO films.

### Pn junction diode fabrication (layered structure)

A W bottom electrode (BE) layer 50 nm thick was first deposited on a SiO_2_ (100 nm)/Si substrate by RF magnetron sputtering. Kapton tape was used as a mask for patterning an NCrGT layer 100 nm thick on a W BE. The NCrGT layer was then crystallized by annealing up to 380 °C. An IGZO layer 40 nm thick was deposited onto the as-deposited and annealed NCrGT by conventional photolithography. The contact area between NCrGT and IGZO can be confined to 6700 µm^2^. A W top electrode (TE) 250 nm thick was deposited *in situ* on the IGZO layer in the same sputtering chamber. W(TE)/IGZO/W(BE) (250 nm/40 nm/50 nm) and W(TE)/NCrGT/W(BE) (250 nm/100 nm/50 nm) device structures were also fabricated by the same process to verify the ohmic conduction behavior between W and IGZO and between W and NCrGT, respectively.

### Hybrid memory/selector device fabrication (Plug-type device)

In this study, we used a substrate with W electrode plugs where the W plug had a square shape and the side length of the square was varied from 34 nm to 1921 nm (see Supplementary Fig. S7). A NCrGT layer 100 nm thick was first deposited on the top of the W-plug using the conventional lithography method. The device was then annealed up to 330 °C to obtain a crystalline phase. Finally, an IGZO layer 40 nm thick and a W TE 250 nm thick were deposited sequentially.

### Measurements

The current-voltage (*I*-*V*) and capacitance-voltage (*C*-*V*) characteristics for all devices were measured using a semiconductor parameter analyzer (B1500A, Keysight). To evaluate the resistive switching properties in the devices, a pulse generator (16440A, Keysight) was used to apply short voltage pulses to the plug-type cells, where the amplitude and pulse width of the pulse voltage applied to the device were confirmed by an oscilloscope (DSO3062A, Agilent Technologies).

## Supplementary Information

Supplementary Information.
