# Supplementary material for: Bidirectional Selector Utilizing Hybrid Diodes for PCRAM Applications
Source: Sci Rep. 2019 Dec 27;9:20209. doi: 10.1038/s41598-019-56768-2 (PMC6934602; doi:10.1038/s41598-019-56768-2)
Supplement: Supplementary file 1 — Supplementary Information. [file 41598_2019_56768_MOESM1_ESM.pdf]

Supplementary information

Bi-directional, Self-selective PCRAM Using Hybrid Diode Characteristics

Yi Shuang[1], *Shogo Hatayama*[1], *Junseop An*[2], *Jinpyo Hong*[3], *Daisuke Ando*[1],

Yunheub Song[2]*, *Yuji Sutou*[1]**

[1]Department of Materials Science, Graduate School of Engineering, Tohoku University, 6-6-11 Aoba-yama, Sendai 980-8579, Japan

[2]Department of Electronics and Communications Engineering, Hanyang University, Seoul 133-791, South Korea

[3]Department of Physics, Hanyang University, Seoul 04763, South Korea

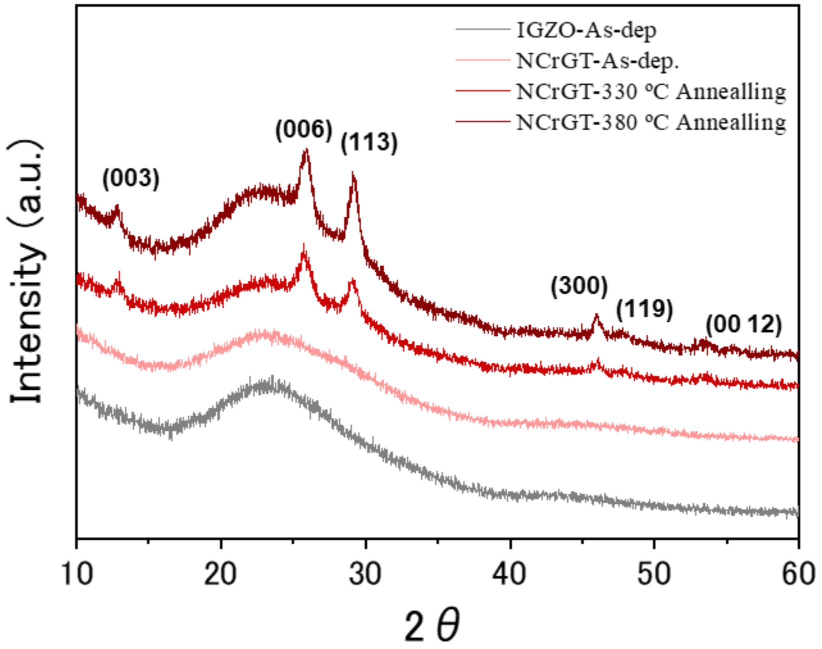

Figure S1. XRD patterns of as-deposited IGZO film and as-deposited and annealed NCrGT films. The annealed NCrGT was annealed up to 330 °C and 380°C, respectively, followed by cooling to room temperature.

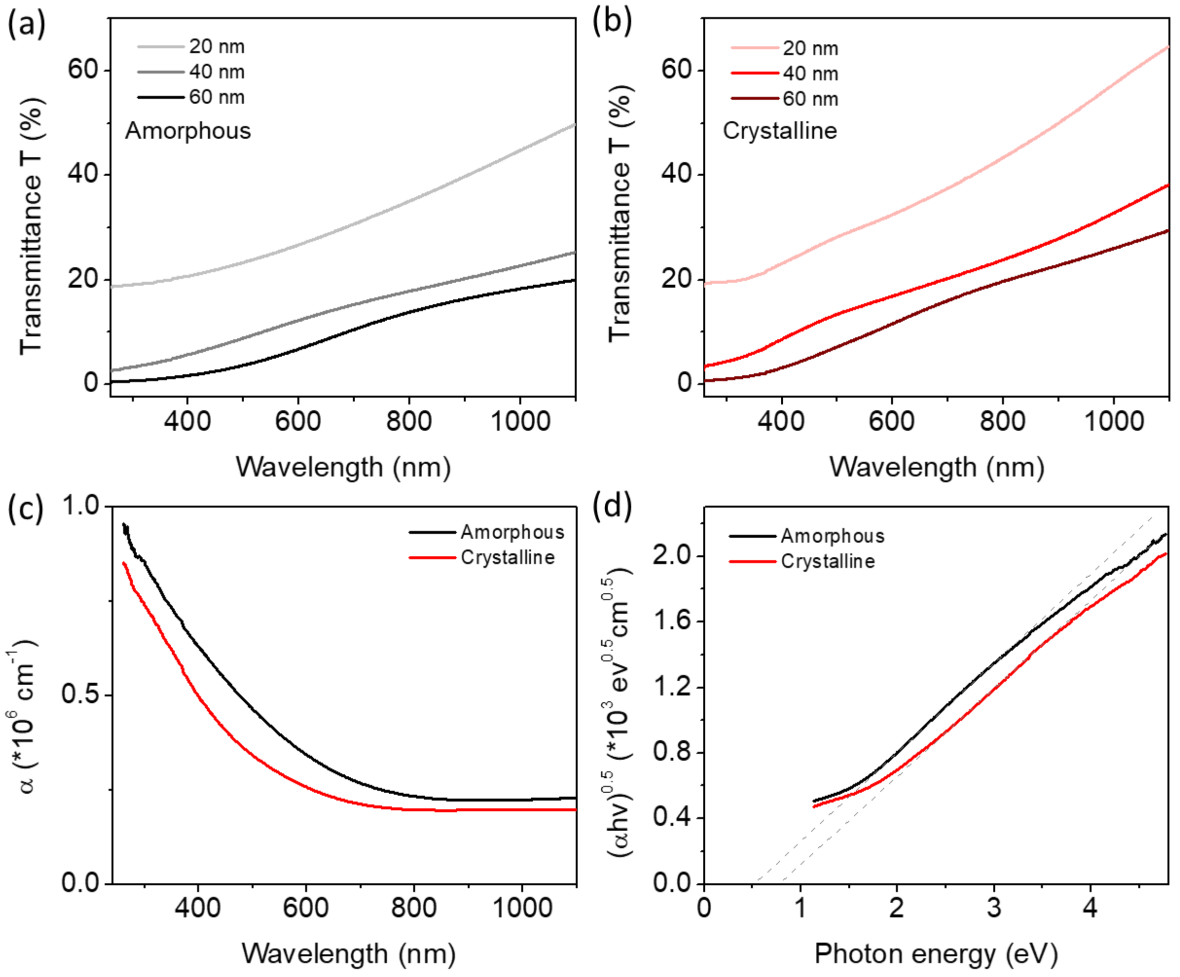

Figure S2. (a) Transmittance spectra for amorphous NCrGT films with various film thickness. (b) Transmittance spectra for crystalline NCrGT films with various film thickness. (c) Absorption coefficient α of NCrGT as a function of wavelength. (d) $(\alpha h\nu)^2$ vs. photo energy ($h\nu$) curves for amorphous and crystalline NCrGT.

The absorption coefficients α can be derived from the transmission and reflection measurements using the following approximation formula:

$$T(\lambda) = \frac{I}{I_0} = (1 - R_1)(1 - R_2)(1 - R_3)(1 - S)\exp{(-\alpha d)} \qquad (1)$$

Where, I_0 is the total intensity incident on film, I is the transmitted light intensity through the film and glass substrate, S is the scattered light that could not reach to detector, R_1, R_2 and R_3 represent the Fresnel reflection at air-film, film-substrate and substrate-air interfaces, respectively. α *is* absorption coefficients, T is transmission and R is reflectance. It is obvious to see from this formula that the reflectance of NCrGT film and multiple reflections and refraction within the interface will have some effects on the calculation of absorption coefficients. Therefore, here we measured the

transmittance of NCrGT films with various thickness (d), d_1=20, d_2=40 and d_3=60 of amorphous and crystalline NCrGT films (Figures S2(a) and S2(b)) and calculated the absorption coefficient based on equation (1). By comparing the transmittance, $T(\lambda 1)$, $T(\lambda 2)$ and $T(\lambda 3)$ at different film thickness, d_1, d_2 and d_3, R_1, R_2, R_3 and S can be eliminated because those are common factors in all thickness, as expressed by equation (2). Then, the absorption coefficient can be finally calculated by the least squares fitting method of equation (3).[1]

$$\frac{T(\lambda_i)}{T(\lambda_j)} = \frac{\exp(-\alpha d_i)}{\exp(-\alpha d_j)} = \exp(-\alpha d_i + \alpha d_j) \text{ (i = 1, 2, 3 and j = 1, 2, 3)} \quad (2)$$

$$\ln(T) = -\alpha d + c \text{ (c is constant)} \quad (3)$$

The value of α as a function of wavelength is shown in Figure S2(c). The optical bandgap of the amorphous and crystalline NCrGT was determined to be 0.5 and 0.8 eV, respectively, as shown in Figure S2(d).

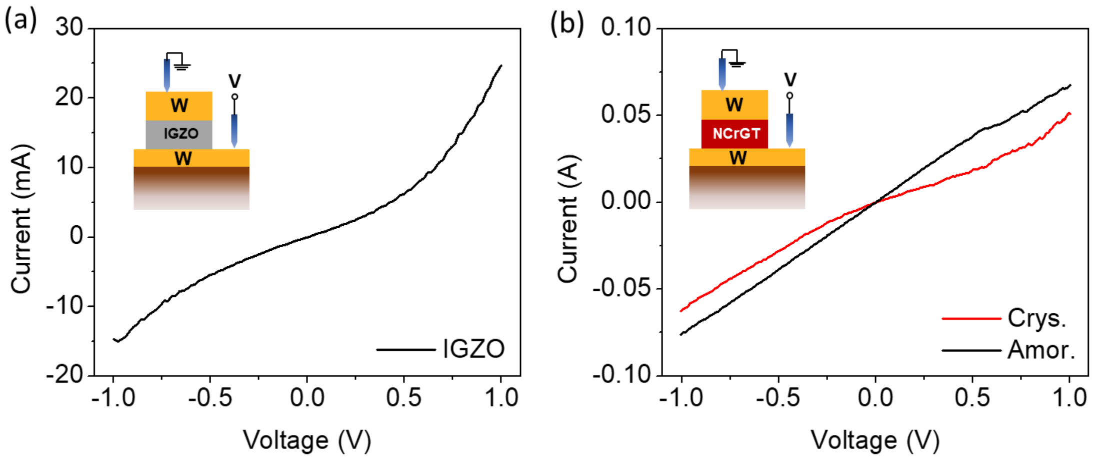

Figure S3. (a) *I*-*V* characteristic of W/IGZO/W layered-structure. (b) *I*-*V* characteristics of W/NCrGT/W layered-structure in both amorphous and crystalline phases.

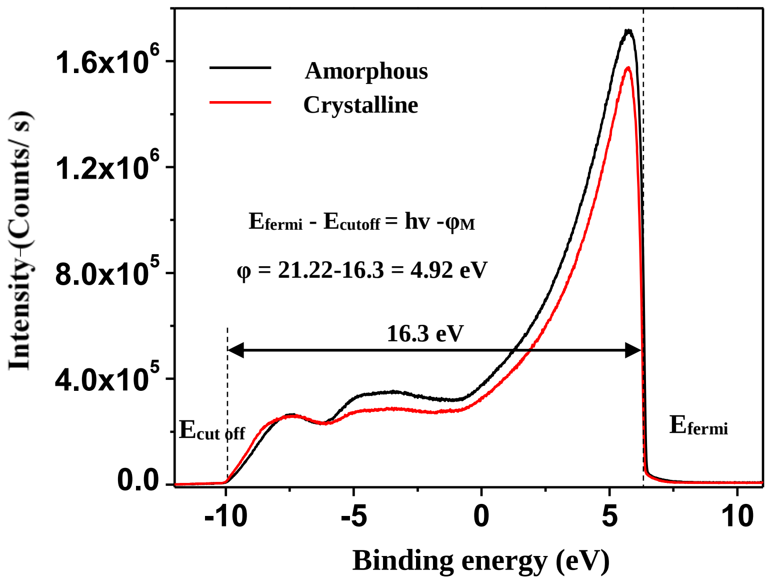

Figure S4. Results of ultraviolet photoelectron spectroscopy (UPS) of amorphous (black) and crystalline (red) phases of NCrGT. The UPS spectra were measured with a He I 21.22 eV excitation line. The position of the E_{cutoff} was determined through Gaussian fitting, and the E_{fermi} was estimated from the quadratic differential method, as shown in Figure S4. The work function φ_M can be obtained to be 4.92 eV for both amorphous and crystalline NCrGT via the expression E_{cutoff}–E_{fermi} = hν–φ_M.

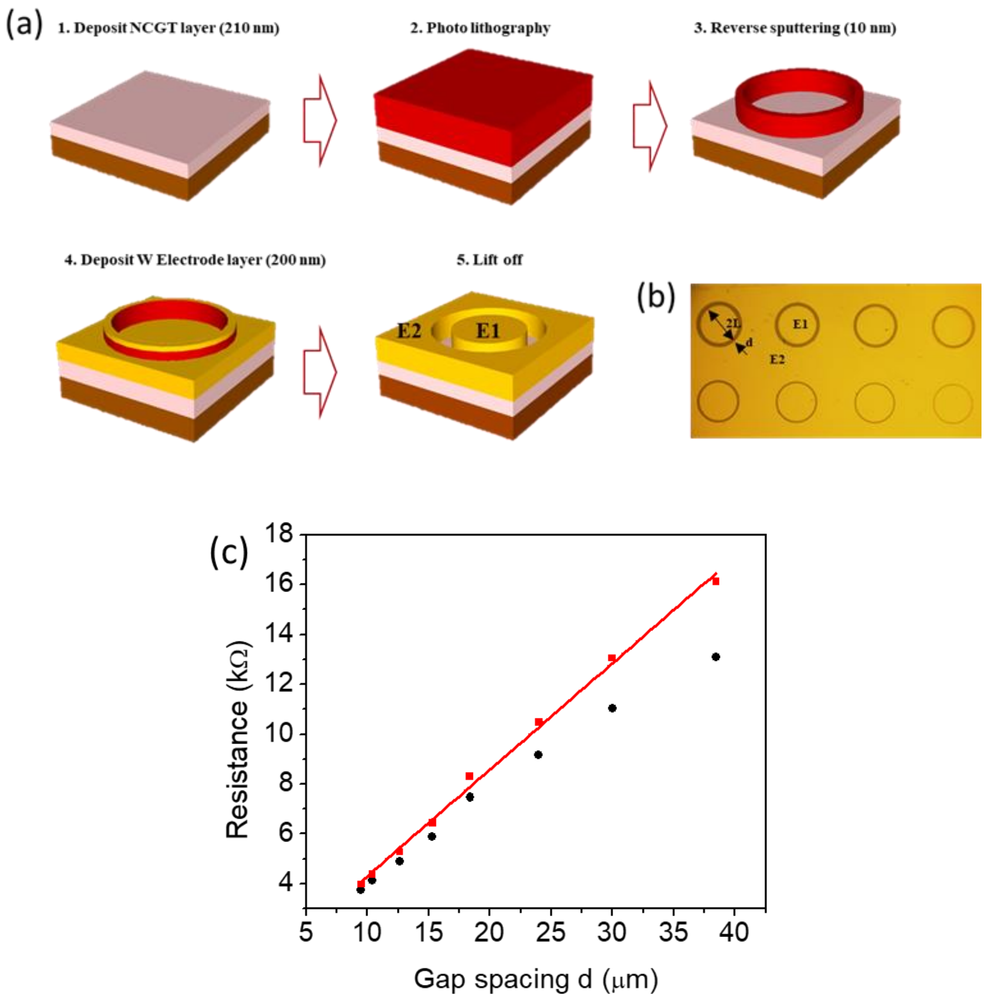

Figure S5. (a) Schematic images of fabrication steps. (b) Optical micrograph image of CTLM pattern, where E1 is the inner electrode with a fixed diameter of 2L (200 µm) and E2 is the outer electrode. The gaps *d* between the inner and outer electrodes were varied from 5 to 40 µm. (c) Measured total resistance (R_T) as a function of gap distance *d* before correction (black dots) and after correction (red dots).

Circular transfer length method (CTLM)

The total resistance (R_T) was characterized by the four-point probe current-resistance method using a semiconductor parameter analyzer. The current flowed from the inner electrode to the outer electrode, and the voltage drop between the two electrodes was measured by another two probes. Eight separate measurements were performed for each CTLM pattern of as-deposited NCrGT. The measured resistance as a function of gap distance *d* is shown in Figure S5(c). R_T between the contacts for each pattern can be expressed as follows:

$$R_T = \frac{R_{sh}}{2\pi L}(d + 2L_t)C$$

and

$$C = \frac{L}{d} ln\,(1 + \frac{d}{L}),$$

where R_{sh} is the sheet resistance of NCrGT on which contact is made, L_t is the transfer length, and *C* is the correction factor. *C* can be calculated from *L* and *d*, which were defined from the optical micrographic image. Then, the measured R_T-*d* curve (black dots) can be linear fitted using *C* (red dots). The transfer length and the sheet resistance were obtained from the intercept of the x-axis and the slope of the linear fitting curve, respectively. The contact resistivity (ρ_c) and resistivity of NCrGT (ρ_{NCrGT}) were calculated using the following equations:

$$\rho_c = R_{sh} * {L_t}^2$$

and

$$\rho_{NCrGT} = R_{sh} * t^2,$$

where *t* is the thickness of the NCrGT thin film. The value of ρ_c was obtained to be $6.4*10^{-6}$ Ω cm^2.

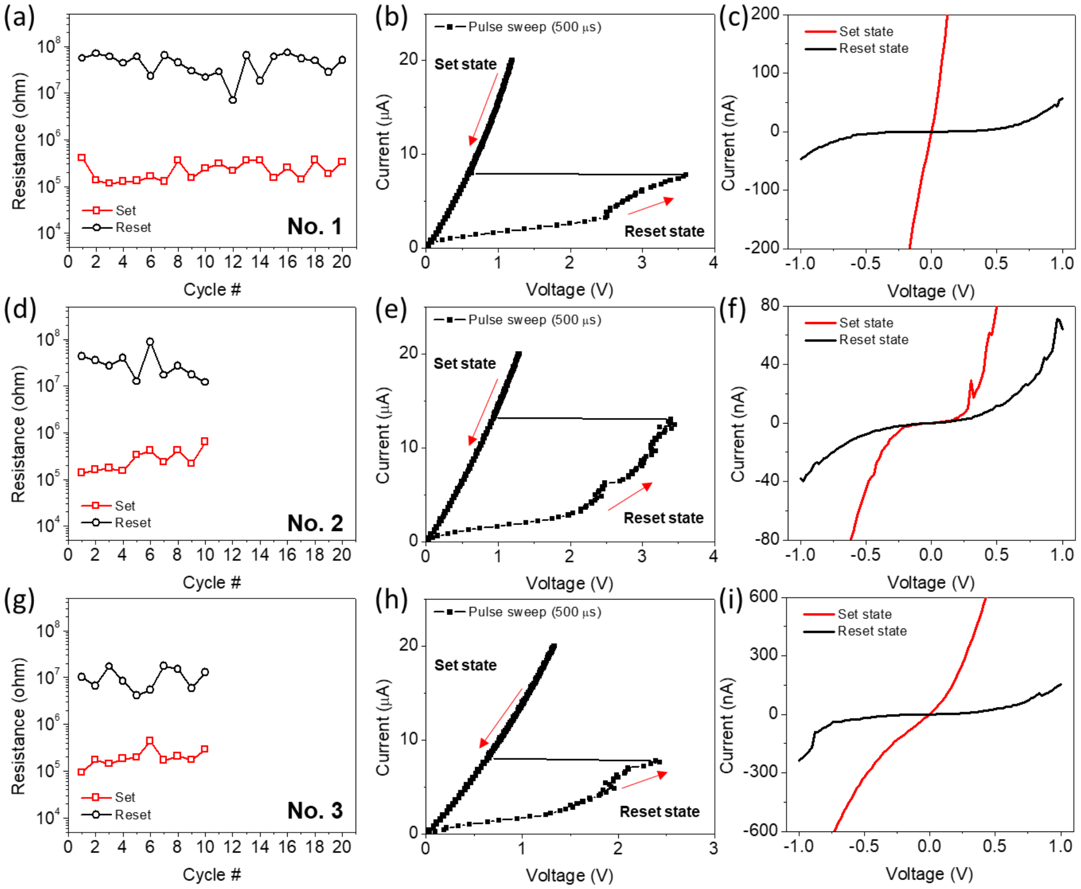

Figure S6. (a), (d) and (g) Cyclic resistive switching memory performance of the device cell 1, 2 and 3 (For set operation, a pulsed current sweep from 0 to 20 μA was applied with pulse width 500 μS; For reset operation, a 100 ns voltage pulse of 3.7 V was applied to the device; The resistance was read at 1 V; All operation procedure were same as expressed in our manuscript). (b), (e) and (h) Threshold switching behavior of the device from reset state of the device cell 1, 2 and 3, respectively. (c), (f) and (i) *I-V* characteristics of reset and set state measured in the last cycle before failure of the device cell 1, 2 and 3, respectively.

The new memory devices were fabricated by same steps as expressed in experimental session, where the TiN plug was used instead of W. It is noteworthy that the work function of TiN is near to W, so the contact resistance contrast between the amorphous and crystalline NCrGT/TiN contact should also be comparable ensuring an enough large set and reset resistance difference in device. Then, the switching behaviors of the fabricated device with the plug size d = 34 nm were measured including cyclic performance, threshold switching curve and I-V characteristics just before the failure and summarized three of the different cells' results in Figure S6. For all cells, the cyclic performance can be improved to some extents while the best cyclic number was still limited to 20 cycles as shown in Figure S6 (a), (d) and (e). Figure S6 (b), (e) and (h) show typical threshold switching behavior of the device from reset state of the device cell 1, 2 and 3, respectively. The

pulsed current applied here is from 0 to 20 μA, which is smaller than W-plug device, indicating the better thermal efficiency of TiN-plug. I-V characteristics of the last cycle of each device in Figure S6 (c), (f) and (i) show a degradation of nonlinearity to some extent, same as in W-plug device.

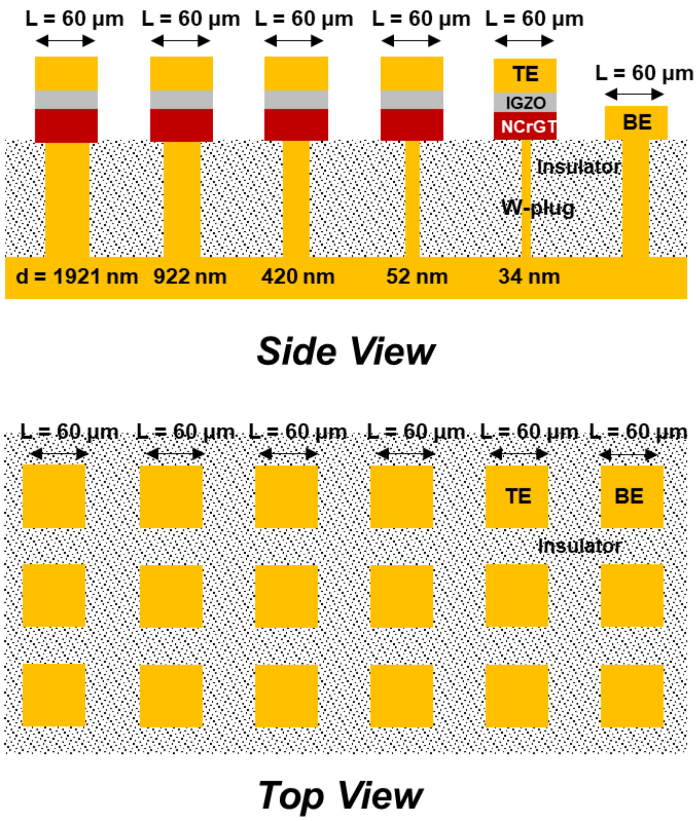

Figure S7. The schematic diagram of the substrate used for the hybrid memory/selector device.

Table S1. Resistivity, carrier density, and mobility of IGZO and NCrGT films.

		Semiconductor type	*Resistivity ρ (Ω·cm)*	*Carrier density n (1/cm³)*	*Mobility μ (cm²/V·s)*
**IGZO**	0.2 sccm O_2	n	15500	1.1×10^{14}	3.62
**NCrGT**	Amor.	p	6.4	1.6×10^{20}	0.020
	Crys.	p	7.6	7.0×10^{19}	0.036

References

1. Ho, H. W. *et al.* Optical response characteristics arising from delocalized electrons in phase change materials. *Acta Mater.* **61,** 1757–1763 (2013).
